# Natural Products and Synthetic Biology: Where We Are and Where We Need To Go

**DOI:** 10.1128/mSystems.00113-19

**Published:** 2019-05-14

**Authors:** Sylvia Kunakom, Alessandra S. Eustáquio

**Affiliations:** a Department of Medicinal Chemistry and Pharmacognosy and Center for Biomolecular Sciences, College of Pharmacy, University of Illinois at Chicago, Chicago, Illinois, USA

**Keywords:** drug discovery, bacterial genomes, biosynthesis, metabolites, structure diversification

## Abstract

The biosynthetic talent of microorganisms has been harnessed for drug discovery for almost a century. Microbial metabolites not only account for the majority of antibiotics available today, but have also led to anticancer, immunosuppressant, and cholesterol-lowering drugs. Yet, inherent challenges of natural products—including inadequate supply and difficulties with structure diversification—contributed to their deprioritization as a source of pharmaceuticals. In recent years, advances in genome sequencing and synthetic biology spurred a renewed interest in natural products. Bacterial genomes encode an abundance of natural products awaiting discovery. Synthetic biology can facilitate not only discovery and improvements in supply, but also structure diversification. This perspective highlights prior accomplishments in the field of synthetic biology and natural products by the scientific community at large, including research from our laboratory. We also provide our opinion as to where we need to go to continue advancing the field.

## Abstract (precis)

The biosynthetic talent of microorganisms has been harnessed for drug discovery for almost a century. Microbial metabolites not only account for the majority of antibiotics available today, but have also led to anticancer, immunosuppressant, and cholesterol-lowering drugs.

## PERSPECTIVE

Drug discovery starts with screening libraries of compounds in biological assays of interest. The input in these screens can be synthetic compounds or natural products: that is, extracts obtained from living systems or pure compounds. Natural products provide privileged scaffolds for drug discovery as they offer structural complexity and physicochemical characteristics that evolved to interact with biological systems. Yet, the natural products themselves rarely become drugs. Rather, they often serve as starting points for drug discovery where natural product derivatives with improved pharmacokinetics and pharmacodynamics properties are then developed into drugs (1, 2).

The structural complexity of natural products is a double-edged sword. Natural products occupy areas of chemical space that are distinct from synthetic molecules, increasing the likelihood of target interaction and hit identification when natural products are included in drug discovery screens (1). At the same time, their structural complexity reduces synthetic tractability, making structure diversification challenging. Moreover, the frequent rediscovery of known compounds and inadequate supply also contributed to deprioritization of natural products research by the private sector starting in the 1990s (3).

In bacteria, biosynthesis of natural products is often encoded in genes that are colocalized in the genome, forming what are referred to as biosynthetic gene clusters (BGCs). About 100,000 bacterial genomes have now been sequenced, and this number will continue to increase as massive genomic and metagenomic efforts are under way. Bioinformatics tools have been developed for automated BGC identification (e.g., see reference 4), leading to an abundance of orphan BGCs for which the encoded compounds are unknown. Given that only a small fraction of known natural products have been connected to BGCs, BGC dereplication (that is, assignment of BGCs to known compounds) helps with genome mining approaches by identifying BGCs that are likely to encode novel natural products (5). BGC dereplication still leaves a wealth of unknown BGCs, and the challenge now is how to prioritize BGCs for discovery (6). Regardless of which criteria we use to select BGCs, genome mining will undoubtedly contribute to natural product discovery in the years to come. We use a broad definition of genome mining here to mean mining genomes not only to identify BGCs and obtain the encoded compounds, but also to connect known compounds to their BGCs.

**FIG 1:**
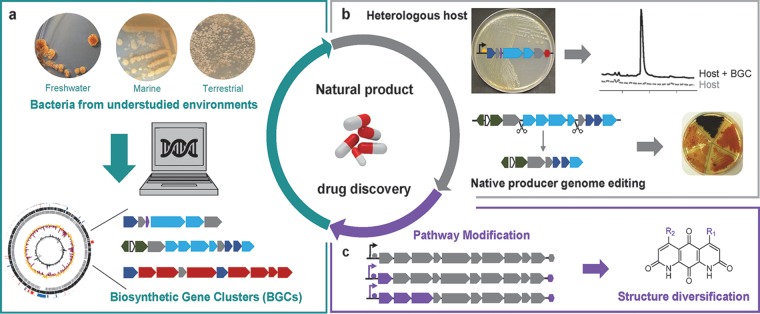
Genomic, genetic, and synthetic biology approaches toward natural product drug discovery. (a) Exploration of bacteria from understudied environments and taxa follows the hypothesis that different selective pressures may lead to the evolution of distinct chemistry. Genomes are sequenced and biosynthetic gene clusters (BGCs) are identified using bioinformatic tools. (b) BGCs of interest are selected. Heterologous expression in suitable hosts can be used to streamline natural product discovery. Genome editing of native producers offers an alternative approach to aid discovery and to study gene and BGC function. (c) Pathway modification via BGC reprogramming contributes to structure diversification of natural products.

One approach we (7) and others (e.g., reference 8) are following to counteract rediscovery and to try to expand the diversity of natural product scaffolds available for drug discovery is to explore understudied environments, the hypothesis being that different environments offer distinct selective pressures that may lead to the evolution of structurally diverse natural products (Fig. 1a). Synthetic biology can facilitate such natural product discovery efforts as it enables going from the DNA sequence in a computer to pure natural products in a bottle without ever having to work with the organisms that encode the information. This can be done by synthesizing BGCs followed by heterologous expression in a host organism (Fig. 1b). Circumventing native producers is relevant because it allows us, for example, to obtain compounds from organisms that have yet to be cultured or to obtain compounds from metagenomic data sets (9). In addition, for cultured organisms, it provides a more streamlined way to increase yields and engineer biosynthesis because native producers may not be genetically tractable.

We would be remiss not to mention that we actually believe that any organism can be eventually genetically engineered and that it is a matter of how much time and effort one wants to put into attempting to develop genetics for organisms that contain BGCs of interest. In fact, we are interested in developing reverse genetics methods (7, 10), as reverse genetics allows us to probe the function of genes in their native setting (Fig. 1b). Reverse genetics also allows us to answer questions such as the function of natural products for the producing organism, questions we are interested in from a basic science perspective. Yet, if the goal is to increase the number of known natural products available to the scientific community in a high-throughput manner, then heterologous expression of BGCs in well-characterized hosts offers the advantage of speed. In this regard, the synthetic biology community seems to be moving away from the idea that “one host fits all” to have hosts tailored to the source of natural products and the compound class. In addition to genetic engineering of native producers, host development is an active area of investigation in our laboratory (Fig. 1b).

Synthetic biology can also complement synthesis efforts and contribute to structure diversification (Fig. 1c). Notable examples include reprogramming of polyketide synthase (PKS) and nonribosomal peptide synthetase (NRPS) modular enzymes. Although the production of natural product derivatives via PKS and NRPS engineering has been demonstrated, engineered systems often suffer from low yields. The Abe group (11) has recently shown that an evolution-inspired engineering strategy can lead to the production of natural product derivatives in good yields. The authors took knowledge regarding the evolution of PKS and NRPS systems into consideration before engineering ring contraction, ring expansion, and alkyl chain diversification of a family of cyclic depsipeptides. Another class of natural products that offer great potential for engineered biosynthesis is ribosomally synthesized and posttranslationally modified peptides (RiPPs). RiPPs have the advantage of being encoded in relatively small BGCs, which greatly facilitates DNA synthesis and synthetic biology efforts toward structure diversification. Most importantly, the broad substrate tolerance of RiPP biosynthetic enzymes allows processing of variable core sequences. A nice demonstration of exploring the promiscuity of RiPP biosynthetic enzymes to discover new inhibitors of drug targets was recently provided by the van der Donk group (12). In this study, the authors coupled a plasmid-encoded library of lanthipeptides with a two-hybrid system to identify inhibitors of a protein-protein interaction critical to the HIV infection cycle. Synthetic biology efforts also benefit from increased knowledge of natural product biosynthesis (10) and the discovery and characterization of enzymes (13) that can be used in pathway construction for engineered biosynthesis.

We envision that in the near future the natural products community will have access to a comprehensive synthetic biology tool box, including computational tools for pathway design, wet lab tools for pathway construction, and an assortment of hosts for BGC expression and compound production. Computational tools are already getting increasingly sophisticated. For example, ClusterCAD, developed by the Keasling group (14), offers an exciting web-based platform to assist with PKS engineering. Moreover, reading DNA is still orders of magnitude cheaper than writing it. Therefore, the field will greatly benefit from cost reduction in DNA synthesis. Recent advances in enzymatic oligonucleotide synthesis (15) may eventually result in new methods that overcome the size limitation (∼200 nucleotides) of current phosphoramidite-based synthesis, which can in turn reduce the reliance on assembly and facilitate the synthesis of BGCs (sizes can range from ∼5 to 200 kb). However, for enzymatic synthesis to become practical, many challenges remain to be overcome, such as improvements in yields and demonstration that large oligonucleotides can be generated with accuracy (15). How long it will take for DNA synthesis costs to significantly drop and catch up with sequencing remains to be seen. Yet, because DNA synthesis is one of the major limitations of synthetic biology, we expect a commensurate effort by the private and academic sectors will lead to innovative approaches and significant cost reduction in the next several years.

Furthermore, efficient *in vitro* and *in vivo* methods to assemble large, repetitive DNA sequences with ease are necessary to leverage the modular logic of PKSs and NRPSs and expand natural product diversity. As we explore untapped microbial taxa, heterologous hosts that work well for these taxa are also necessary. Additionally, because drug discovery often requires chemical transformations that are not necessarily found in nature, directed evolution (16) of enzymes and pathways is expected to greatly contribute to synthetic biology efforts aimed at structure diversification of natural products.

Synthetic biology is still a trial-and-error endeavor relying on build-test-learn cycles. We still need to learn how to design pathways that work efficiently. Or do we? We made the mistake before to presume that we could design enzymes, or, in other words, that we could predict function based on sequence. Frances Arnold (16) and others showed us that directing evolution via random mutagenesis and selection/screening is actually the more rational way to go, because we are, as of yet, unable to predict beneficial mutations. Could the same be true for the design of chimeric, modular enzymes or for biosynthetic pathway design? In any case, we expect that evolution-inspired approaches such as those recently reported by the Abe group (11) have the best chances of leading to the desired function, and we may one day learn how to design by observing evolution.
